# Sex-specific differences of cardiopulmonary fitness and pulmonary function in exercise-based rehabilitation of patients with long-term post-COVID-19 syndrome

**DOI:** 10.1186/s12916-024-03658-8

**Published:** 2024-10-08

**Authors:** René Garbsch, Hendrik Schäfer, Mona Kotewitsch, Johanna M. Mooren, Melina Waranski, Marc Teschler, Katalin Vereckei, Gereon Böll, Frank C. Mooren, Boris Schmitz

**Affiliations:** 1 Department of Rehabilitation Sciences, Faculty of Health, University of Witten/Herdecke, Witten, Germany; 2 Center for Medical Rehabilitation, DRV Clinic Königsfeld, Klinik Königsfeld, Holthauser Talstraße 2, 58256 Ennepetal, Germany

**Keywords:** SARS-CoV-2, Long-COVID, Exercise-based rehabilitation, Severe Acute Respiratory Syndrome, Fatigue

## Abstract

**Background:**

Post-COVID-19 Syndrome (PCS) entails a spectrum of symptoms, including fatigue, reduced physical performance, dyspnea, cognitive impairment, and psychological distress. Given the effectiveness of exercise-based rehabilitation for PCS, this study examined the efficacy of rehabilitation for PCS patients, focusing on sex-specific differences.

**Methods:**

Prospective cohort study during inpatient rehabilitation. Cardiopulmonary exercise testing and spirometry were performed at admission and discharge. Questionnaires were used to assess fatigue, health-related quality of life, wellbeing, and workability for up to 6 months.

**Results:**

145 patients (36% female, 47.1 ± 12.7 years; 64% male, 52.0 ± 9.1 years; *p* = 0.018) were referred to rehabilitation 262.0 ± 128.8 days after infection (female, 285.5 ± 140.6 days; male, 248.8 ± 112.0 days; *p* = 0.110). Lead symptoms included fatigue/exercise intolerance (81.4%), shortness of breath (74.5%), and cognitive dysfunction (52.4%). Women presented with higher relative baseline exercise capacity (82.0 ± 14.3%) than males (68.8 ± 13.3%, *p* < 0.001), but showed greater improvement in submaximal workload (*p* = 0.026). Men exhibited higher values for FEV1, FEV1/VC, PEF, and MEF and lower VC at baseline (*p* ≤ 0.038), while FEV1/VC improvement more in women (*p* = 0.027). Higher baseline fatigue and lower wellbeing was detected in women and correlated with impaired pulmonary function (*p* < 0.05). Disease perception including fatigue, health-related quality of life, wellbeing and workability improved with rehabilitation for up to six-month.

**Conclusions:**

Rehabilitation improves cardiopulmonary fitness, pulmonary function and disease burden in women and men with long-term PCS. Women with PCS may benefit from intensified respiratory muscle training. Clinical assessment should include cardiopulmonary exercise testing and pulmonary function tests and fatigue assessments for all PCS patients to document limitations and tailor therapeutical strategies.

**Supplementary Information:**

The online version contains supplementary material available at 10.1186/s12916-024-03658-8.

## Background

Post-COVID-19 Syndrome (PCS), often also referred to as post-acute sequelae of COVID-19, emerges subsequent to an acute infection with the SARS-CoV-2 virus (COVID-19 infection). PCS is defined by the persistence of symptoms for a duration longer than 12 weeks from the onset of infection and/or the emergence of new symptoms within this timeframe [1]. The characteristics of PCS are still under investigation, and while recent guidelines propose specific criteria for PCS diagnosis [1, 2], PCS is to some extent marked by diagnostic ambiguity due to the complexity of its symptomatology and the absence of definitive diagnostic tools [3]. PCS can be characterized as a multisystemic disorder, with the most prevalent symptoms encompassing (chronic) fatigue, diminished physical performance, muscular weakness and pain, dyspnea, cognitive impairments and alterations of the autonomous nervous system, as well as mental and psychological distress [2–6]. The severity of symptoms varies considerably among patients, ranging from mild impairment to significant restrictions in daily activities, potentially including (temporary) partial or complete incapacity to work [2, 3]. A COVID-19 infection may instigate various processes that remain incompletely understood also in terms of their contributions to onset and severity of PCS. Potential factors involve endothelial dysfunction, a "cytokine storm" linked to excessive oxidative stress and mitochondrial dysfunction affecting numerous organs and subcellular structures, as well as the microvasculature, among others [7, 8]. Even if PCS appears to be more frequent after more severe acute infection and in patients with pre-existing medical conditions [9], it may also occur after mild infection, while individual risk factors for PCS are subject to ongoing debate [1, 10]. While the majority of patients undergo a gradual recovery without specific treatment, there is a substantial demand for effective medical rehabilitation, especially for patients with persistent PCS [1, 4]. Even though larger individual studies and a recent meta-analysis suggested overall beneficial effects on physical performance and PCS-specific symptoms, studies applying gold standard cardiopulmonary exercise testing (CPET) to document improvement of physical fitness are scarce [11–15]. In addition, exercise-based programs lasting 2–12 weeks induced significantly higher quality of life, reduced fatigue, and less depression at least in patients recently recovered from an acute COVID-19 infection, with often unreported long-term effects [11, 13, 14, 16]. While risk factors predisposing COVID-19 patients for the development of PCS are still under investigation [9], female sex has been suggested as one significant contributor to increased occurrence of persistent symptoms [17, 18]. However, sex-specific differences in PCS patients referred to rehabilitation have so far not been reported and it is currently unknown if women and men benefit to the same extend from exercise-based rehabilitation. Thus, the objective of this study was to compare the effects of exercise-based rehabilitation on cardiopulmonary exercise capacity, pulmonary function, and (health related) outcomes including fatigue, quality of life, wellbeing, depression and workability between long-term female and male PCS patients.

## Methods

### Study design and patients

A prospective observational cohort study of PCS patients referred for inpatient medical rehabilitation at Clinic Königsfeld, Germany was performed between August 2021 and July 2023. Inclusion criteria were a history of (at least one) COVID-19 infection (positive PCR test at the time of infection), and ongoing or newly expressed performance deficits lasting for at least 3 months prior to recruitment. In total, 145 PCS patients (*N* = 52 female, *N* = 93 male) were included and full clinical assessment including symptom-limited CPET and pulmonary function tests were performed at enrollment and before discharge. Validated questionnaires were applied at enrollment, discharge, and 6-months follow-up to assess disease perception and workability. Performance deficits were documented according to the recent consensus statement, with the cluster of lead symptoms including fatigue/exercise intolerance, shortness of breath, and cognitive dysfunction impairing activity of daily living and everyday functioning [5]. History of comorbidities and current medication were documented, and blood samples for full blood count were drawn on the day of admission.

### Ethical approval

The study was approved by the local ethical review committees (Ethik-Kommission Universität Witten/Herdecke; reference number 159/2021) and conformed to the Declaration of Helsinki. Written informed consent was obtained from all participants.

### Medical rehabilitation concept

Patients received individual medical rehabilitation including a combination of active, cognitive and passive therapies. Active therapies consisted mainly of physical therapies with a combination of strength, endurance and respiratory training, such as exercise in groups, aerobic ergometer training (see below), medical training therapy, aqua fitness, walking and circuit training (whole-body strength endurance and aerobic endurance training with light equipment or body weight) as well as inspiratory muscle training. Predominantly cognitive therapies consisted of disease education, psychological counseling, nutrition education, stress management and coping strategies as well as occupational therapy and brain/memory training. Predominantly passive therapies consisted of relaxation therapies such as muscle relaxation, light or heat therapy and massages. Therapies were prescribed by doctors according to physical performance status and lead symptoms at admission, implemented and adapted by experienced therapists. A large proportion of active therapies consisted of aerobic ergometer training (Ergoline Select 100, Ergoline GmbH, Bitz, Germany), which patients performed either as continuous training (50% of maximal workload) or interval training (load, 60% for 100 s; recovery, 30% for 48 s) matched for workload and time (both 18 min per session) as described [15]. Intensity was guided by individual training pulse (calculated at the beginning of rehabilitation using the Karvonen formula) and perceived intensity of the load, resulting in adjusted workloads. Other active therapies were individually adjusted in form of increased intensity (walking, aqua fitness) or more repetitions per set/session (resistance training, circuit training). Prescription was individualized and adjusted based on therapists’ evaluation with no difference for female and male patients. Data on rehabilitation procedures including prescriptions of therapeutic actions and participation was recorded. Minutes of exercise were recorded and converted into metabolic equivalents (MET) [19]. During in-patient rehabilitation, patients were provided with a full diet, based on the recommendatations of the German Nutrition Society.

### Cardiopulmonary exercise testing (CPET)

Symptom-limited ergometer testing with continuous breath-by-breath respiratory gas exchange analysis was conducted following manufacturer’s guidelines (Ergostic, Amedtech, Aue, Germany) as part of the standard clinical diagnostic procedure upon admission and within three days prior to discharge. Expiratory flow measurements were performed using a mass flow sensor, calibrated with a known concentration gas mixture prior to each assessment. The initial assessment of physical fitness in PCS patients was conducted through a cardiac stress electrocardiogram, and a tailored ramp protocol was selected for CPET based on the results as follows: 1. for low performance (< 100W), initiation at 20W with increments of 15W every 2 min; 2. for medium performance (100-125W), initiation at 20W with increments of 20W every 2 min; 3. for moderate performance (> 125W), initiation at 25W with increments of 25W every 2 min. The same protocol was used at admission and before discharge for each individual patient. Participants were instructed to achieve a rating of perceived exertion (RPE) of ≥ 8 on the 0–10 Borg Scale during the test. Continuously recorded variables included workload (W), heart rate (HR), oxygen consumption (VO_2_), carbon dioxide production (VCO_2_), and minute ventilation (VE). Peak VO_2_ was defined as the maximum oxygen uptake reported relative to bodyweight and as a percentage of reference (predicted value corrected for sex, age, and body surface area) for comparability. VO_2_ at the ventilatory thresholds (VT1, first ventilatory threshold; VT2, second ventilatory threshold) was determined using Ergostic software and visually confirmed using both the V-slope (VT1) and V’E-Slope (VT2) method as well as the ventilatory equivalent method (VE/VO_2_) according to Wasserman/Hansen and using the respective reference values for comparison [20]. The oxygen pulse was calculated as the VO_2_/HR ratio.

### Pulmonary function

Patients performed standard spirometry measurements (Ergostic) before each CPET to assess vital capacity (VC), forced expiratory volume in 1 s (FEV1), peak expiratory flow (PEF) and maximum expiratory flow of forced expiratory vital capacity (MEF). Maximum inspiratory pressure (MIP) was tested at admission and before discharge according to manufacturer’s instructions (PowerBreathe KH2, HaB GmbH, Winsen a.d. Luhe, Germany).

### Assessment of disease perception

The assessment of disease burden and its functional impact on daily life, including fatigue, was conducted at three separate time points: enrollment, discharge, and after six months follow-up using established and validated questionnaires. Fatigue in PCS was quantified using the Multidimensional Fatigue Inventory (MFI-20) as described previously [21]. The MFI-20 provides an aggregate score and two subscales pertaining to physical and mental fatigue. Scores range from 0 to 100, with higher values indicating elevated levels of fatigue. Health-related quality-of-life was assessed using the SF-36 questionnaire, with eight domains: physical functioning, physical role, bodily pain, general health, vitality, social functioning, emotional role, and mental health. The SF-36 generates two scores, a Physical Component Score and a Mental Component Score, both ranging from 0 to 100, with higher scores signifying a more favorable functional status [22]. The WHO-5 questionnaire was employed to assess the general level of well-being, with scores ranging from 0 to 25, where greater scores indicating increased well-being [23]. The assessment of anxiety and depression severities was done using the Hospital Anxiety Depressions Scale (HADS), administered at enrollment and prior to discharge. The subscales are graded as follows: 0–7 = ‘normal’, 8–10 = ‘mild’, 11–14 = ‘moderate’, and 15–21 = ‘severe’. The evaluation of work ability was performed at enrollment and after six months of discharge using the Work Ability Index (WAI) questionnaire. The WAI comprises various subscales including present working capacity, job-related ability, diagnosed pathologies, reduction in working capacity due to illness, sick leave over the past year, personal expectations regarding future work skills, and psychological conditions/resources [24]. The WAI score may be categorized as: low (7-27), moderate (28-36), good (37–43), or excellent (44–49). All questionnaires used had been validated in German populations [25–28].

### Statistical analysis

CPET data and spirometric measurements were available from all included patients at both time points. Data was analyzed using SPSS (V.28, IBM, Armonk, USA) and GraphPad Prism (V.10, GraphPad Software, Boston, USA). Constant variables are expressed as mean ± standard deviation (SD) or 95% confidence interval (CI) or median (range) as indicated. Categorical variables are presented as n (%). Non-normal distribution was tested using skewness and kurtosis. Differences between women and men over time were analyzed using 2-way repeated measures ANOVA or mixed-effects model, unpaired two-sided t-test or Mann–Whitney U test if indicated. Chi-square test was used for categorical variables. Analysis of sex-related differences was based on percent-predicted values (percentage of reference, corrected for sex, age, and body surface area [calculated from weight and height]); within-group comparison was performed using absolute values. ANCOVA was performed to adjust for confounding effects of age, body mass index, comorbidities (especially diseases of the circulatory system) and differences in baseline values between women and men. Responder analysis was performed for VO_2_ at VT1 and peak exercise as described using the typical error (TE) method and the following equation: $\text{TE}=\text{SD}{\text{diff}} \, /\sqrt{2}$, where SD_diff_ is calculated as the difference between the variance (SD) of two repeated measures [29]. Responders were defined as participants who demonstrated an increase greater than 2 × TE away from zero. Spearman rank correlation analyses were performed to investigate correlations between physical exercise capacity, pulmonary function and disease perception. Power analysis (G*Power, V3.1.9.7, Düsseldorf, Germany) revealed that a sample size of 147 would be required to detect an overall improvement in VO_2peak_ with a power of 1-beta = 0.95 at alpha = 0.05 (pre-post-comparison, paired two-sided t-test) given an effect size of 0.3 (based on own preliminary data). Statistical significance was accepted at *p* < 0.05.

## Results

### Baseline characteristics

#### Clinical picture and comorbidities

**Fig. 1:**
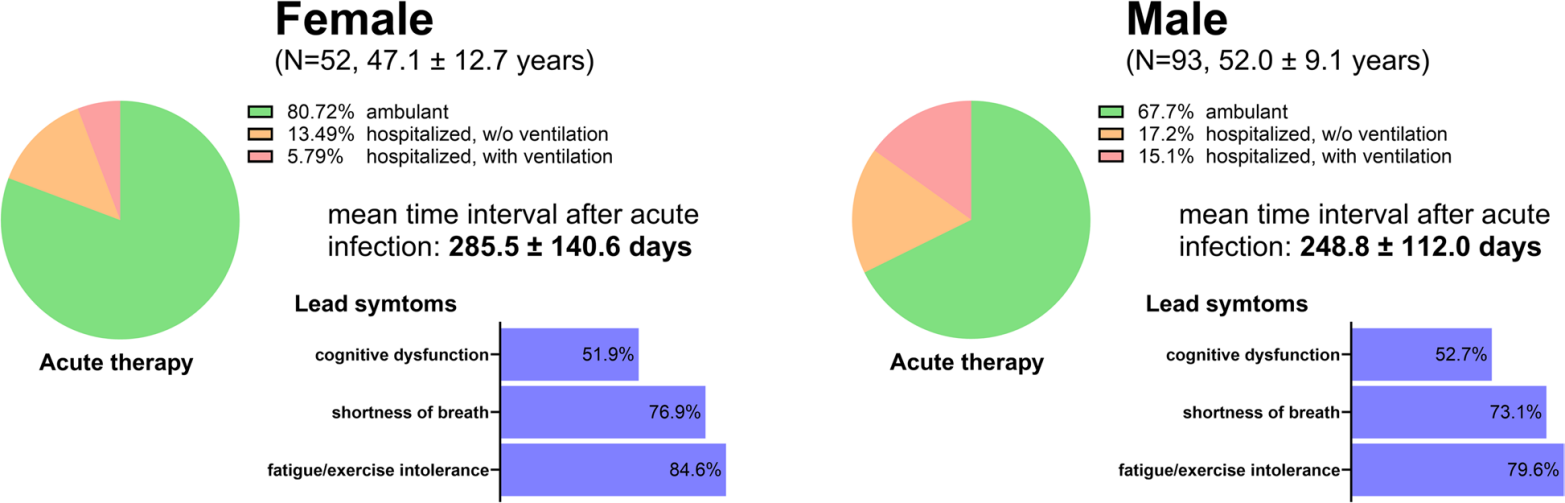
Characteristics of female and male patients with Post-COVID-19 syndrome (PCS). PCS patients were referred to rehabilitation 262.0 ± 123.8 days after acute infection (no difference between women and men, *p* = 0.110). At admission, affected women were significantly younger than men (*p* = 0.018). During the acute phase of infection, women had less often been hospitalized (*p* = 0.092), and need for ventilation during acute care was lower (*p* = 0.096). Women and men did not differ in lead symptoms at the onset of rehabilitation (all *p* ≥ 0.454; multiple naming possible). Data is presented as mean ± SD or %. Between-group comparison was performed using unpaired two-sided t-test or Chi-square test for categorical variables

PCS Patients (36% women) were referred to rehabilitation with a mean age of 50.2 ± 10.7 years (female, 47.1 ± 12.7; male, 52.0 ± 9.1; Fig. 1) and a mean Body Mass Index (BMI) of 30.4 ± 5.9 kg/m^2^ (female, 28.7 ± 6.1; male, 31.3 ± 5.6). The mean time interval between the first infection and start of medical rehabilitation was 262.0 ± 123.8 days (female, 285.5 ± 140.6 days; male, 248.8 ± 112.0 days; *p* = 0.110). No significant differences between female and male patients were detected with respect to lead symptoms (all *p* ≥ 0.458). Overall, fatigue/exercise intolerance was the most prevalent lead symptom observed in 81.4% of patients, followed by shortness of breath (74.5%), and cognitive dysfunction (52.4%) (Fig. 1). During the acute phase of infection, 72.4% of patients had received ambulant care or acute care at home (female, 80.7%; male, 67.7%), while 27.6% of patients required in-hospital care (female, 19.3%; male, 32.4%; *p* = 0.092). The need for ventilation during hospitalization was lower in women (not significant, *p* = 0.096). Patients reported a high frequency of endocrine, nutritional, and metabolic disorders, as well as circulatory system disorders (Table 1), the latter being more frequent in men (*p* = 0.031). Accordingly, medication profiles exhibited variations between female and males. Results of blood analyses were within the reference range (data not shown). Overall, 33.1% were ever smoker (female, 28.8%; male, 35.5%). No correlations between time after acute infection and baseline exercise capacity, pulmonary function and perceived disease burden were detected (Additional file 1: Figure S1).

**Table 1:** Comorbidities and clinical data

	**Overall *N* = 145**	**Female *N* = 52**	**Male *N* = 93**	***p*-value**
**Comorbidities**				
**Endocrine, nutritional or metabolic diseases**	109 (75.2)	37 (71.2)	72 (77.4)	0.402
*Obesity*	72 (49.7)	26 (50.0)	46 (49.5)	0.950
*Hyperlipidemia*	19 (13.1)	5 (9.6)	14 (15.1)	0.352
*Type 2 diabetes mellitus*	16 (11.0)	4 (7.7)	12 (12.9)	0.337
*Hypothyroidism*	13 (9.0)	3 (5.8)	10 (10.8)	0.314
*Other*	22 (15.2)	6 (11.5)	16 (17.2)	0.362
**Diseases of the circulatory system**	92 (63.4)	27 (51.9)	65 (69.9)	**0.031**
*Arterial hypertension*	73 (50.3)	21 (40.4)	52 (55.9)	0.073
*Paroxysmal tachycardia*	21 (14.5)	1 (1.9)	4 (4.3)	0.452
*Atrial fibrillation*	9 (6.2)	5 (9.6)	4 (4.3)	0.203
*Pulmonary embolism*	7 (4.8)	2 (3.8)	5 (5.4)	0.680
*Venous thrombosis*	6 (4.1)	4 (7.7)	2 (2.2)	0.108
*Coronary artery disease*	6 (4.1)	2 (3.8)	4 (4.3)	0.895
*Other*	22 (15.2)	4 (7.7)	18 (19.4)	0.060
**Diseases of the musculoskeletal system and connective tissue**	52 (35.9)	18 (34.6)	34 (36.6)	0.815
**Diseases of the nervous system**	34 (23.4)	13 (25.0)	21 (22.6)	0.742
*Migraine/ headache*	6 (4.1)	3 (5.8)	3 (3.2)	0.461
*Other*	29 (20.0)	10 (19.2)	19 (20.4)	0.863
**Mental and behavioral disorders**	34 (23.4)	14 (26.9)	20 (21.5)	0.460
*Depressive/ adjustment disorders*	21 (14.5)	10 (19.2)	11 (11.8)	0.224
*Other*	15 (10.3)	4 (7.7)	11 (11.8)	0.433
**Diseases of the respiratory system**	18 (12.4)	8 (15.4)	10 (10.8)	0.417
**Diseases of the digestive system**	16 (11.0)	4 (7.7)	12 (12.9)	0.337
**Neoplasms**	8 (5.5)	2 (3.8)	6 (6.5)	0.510
**Medication**^**a**^				
**Beta blocker**	50 (34.5)	17 (32.7)	33 (35.5)	0.734
**Anticoagulant**	32 (22.1)	9 (17.3)	23 (24.7)	0.301
**Analgesic**	31 (21.4)	16 (30.8)	15 (16.1)	**0.039**
**AT-II receptor blocker**	30 (20.7)	5 (9.6)	25 (26.9)	**0.014**
**ACE inhibitor**	27 (18.6)	6 (11.5)	21 (22.6)	0.101
**Calcium channel blocker**	27 (18.6)	3 (5.8)	24 (25.8)	**0.003**
**Diuretic**	27 (18.6)	6 (11.5)	21 (22.6)	0.101
**Statin**	26 (17.9)	4 (7.7)	22 (23.7)	**0.016**
**Glucocorticoid**	23 (15.9)	9 (17.3)	14 (15.1)	0.722
**Antidepressant**	14 (9.7)	6 (11.5)	8 (8.6)	0.566
**Diabetes medication**	8 (5.5)	0 (0)	8 (8.6)	**0.030**
**Antiarrhythmic**	2 (1.4)	1 (1.9)	1 (1.1)	0.675

Data is presented as n (%). Between-group comparison was performed using unpaired two-sided t-test, Mann–Whitney U test, or Chi-square test. “Other” refers to diagnoses of the respective ICD-10-category present in < 4% of patients

^a^Medication at admission. For the majority of patients, medication remained unchanged, adjustment was only required in few cases

#### Exercise capacity and pulmonary function

**Fig. 2:**
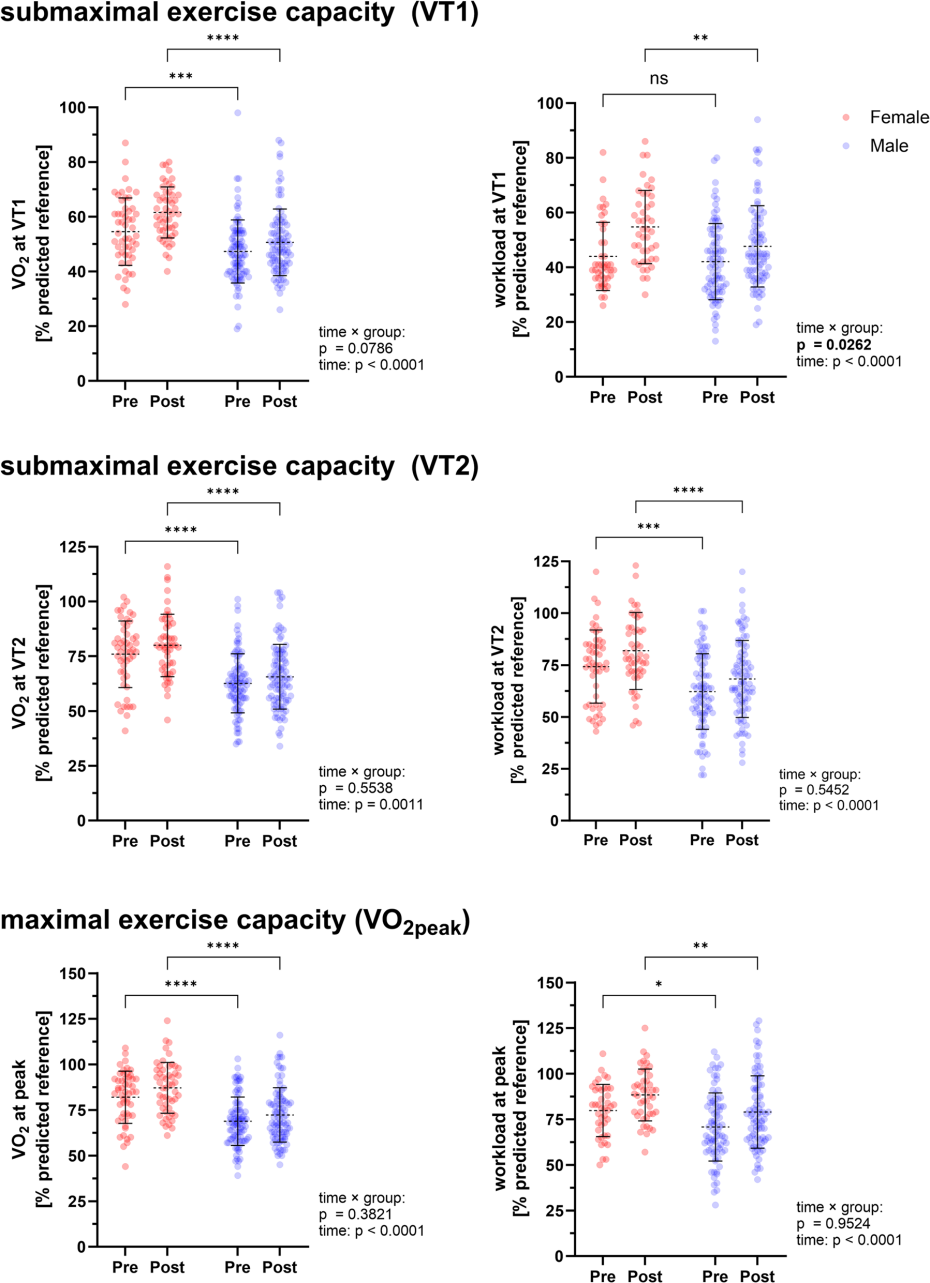
Female and male patients with Post-COVID-19 syndrome (PCS) differ in submaximal and maximal exercise capacity. Female PCS patients (*n* = 52) showed higher relative submaximal and maximal exercise capacity determined by oxygen uptake (cardiopulmonary exercise testing, CPET) compared to male PCS patients (*n* = 93) at start of rehabilitation. While females and males showed an overall improvement of exercise capacity in response to rehabilitation (significant time effect), female patients showed a significantly greater improvement in submaximal workload (watt @ VT1) compared to males (significant time × group interaction). Pre: at admission, Post: at discharge. Data is presented as mean ± SD, each data point represents an individual measurement. Differences between groups over time were performed using two-way repeated measures ANOVA of percent predicted values (reference values) corrected for sex, age and body surface area. * *p* ≤ 0.05, ** *p* ≤ 0.01, *** *p* ≤ 0.001, **** *p* ≤ 0.0001

At baseline, PCS patients exhibited a significant limitation in submaximal and peak exercise capacity at 12.3 ± 3.4 ml/min/kg and 18.0 ± 4.3 ml/min/kg equal to 49.9 ± 12.3% and 73.6 ± 15.0% of predicted reference, respectively. Of note, women presented with a significantly higher relative peak exercise capacity (VO_2_, ml/min/kg) of 82.0 ± 14.3%, compared to men with 68.8 ± 13.3% (*p* ≤ 0.001; Fig. 2; Table 2). In addition, women had significantly higher heart rate and O_2_-pulse at submaximal exercise, as well as higher peak heart rate, O_2_-pulse and respiratory minute ventilation and reached higher workload compared to men using relative reference values for adjusted comparison (all *p *≤ 0.018). Restrictions in pulmonary function were mainly reflected in reduced expiratory flow variables (Table 3) and maximum inspiratory pressure (MIP). Men exhibited higher values for FEV1, FEV1/VC, PEF and maximum expiratory flow (MEF25-75) (all *p* ≤ 0.038) and lower vital capacity (VC) at baseline compared to women (*p* = 0.019). The threshold for inspiratory weakness (≥ 80 cmH_2_O) was reached in 66.2% of PCS patients with significant difference between women and men (female, 78.8%; male 59.1%; *p* = 0.038).

**Table 2:** Changes in exercise capacity assessed by cardiopulmonary exercise test (CPET)

	**Overall *N* = 145**		***p*-value time**	**Female *N* = 52**		**Male *N* = 93**		***p*-value group**
	**absolute**	**% predicted**		**absolute**	**% predicted**	**absolute**	**% predicted**	
**Resting**								
**Heart rate, beat·min**^**−1**^								
T0	88.1 ± 11.8	n.a		90.6 ± 11.9	n.a	86.8 ± 11.6	n.a	0.062
T1	83.6 ± 11.0			86.4 ± 12.2		82.1 ± 10.1		0.030
∆	-4.5 ± 10.5		**0.001**	-4.2 ± 9.5		-4.7 ± 11.0		0.768
**O**_**2**_** pulse, ml·beat**^**−1**^								
T0	6.7 ± 1.8	n.a		5.7 ± 1.3	n.a	7.3 ± 1.8	n.a	0.001
T1	6.9 ± 1.5			5.8 ± 1.1		7.5 ± 1.4		0.001
∆	+ 0.1 ± 1.4		0.259	+ 0.1 ± 0.9		+ 0.2 ± 1.6		0.617
**Ventilatory equivalent O**_**2**_** (VE/VO**_**2**_**)**								
T0	27.9 ± 5.5	n.a		27.2 ± 4.7	n.a	28.2 ± 5.8	n.a	0.322
T1	27.8 ± 6.0			27.5 ± 4.2		28.0 ± 6.8		0.655
∆	-0.0 ± 6.2		0.944	+ 0.3 ± 5.0		-0.2 ± 6.8		0.659
**Ventilatory equivalent CO**_**2**_** (VE/VCO**_**2**_**)**								
T0	33.7 ± 5.2	n.a		33.2 ± 3.7	n.a	33.4 ± 5.9	n.a	0.824
T1	34.0 ± 4.4			33.6 ± 3.5		34.3 ± 4.8		0.374
∆	+ 0.6 ± 4.7		0.098	+ 0.3 ± 3.0		+ 0.8 ± 5.4		0.560
**Ventilatory threshold 1 (VT1)**								
**Workload, watt**								
T0	73.8 ± 27.9	43.4 ± 15.1		60.2 ± 18.8	44.0 ± 12.5	81.4 ± 29.3	43.1 ± 16.4	0.737
T1	86.0 ± 27.0	50.6 ± 15.7		74.1 ± 20.2	54.7 ± 13.4	92.7 ± 28.1	48.4 ± 16.4	0.030
∆	+ 12.3 ± 23.1	+ 7.2 ± 13.1	**0.001**	+ 14.0 ± 15.7	+ 10.7 ± 11.8	+ 11.3 ± 26.3	+ 5.3 ± 13.4	**0.026#**
**Heart rate, beat·min**^**−1**^								
T0	108.8 ± 15.9	65.4 ± 8.8		111.0 ± 16.9	67.9 ± 8.7	107.6 ± 15.2	64.0 ± 8.5	0.009
T1	107.8 ± 19.2	64.8 ± 10.7		112.3 ± 24.4	68.7 ± 13.1	105.3 ± 15.2	62.7 ± 8.5	0.004
∆	-1.0 ± 17.4	-0.5 ± 10.7	0.493	+ 1.3 ± 20.4	+ 0.8 ± 13.0	-2.3 ± 15.5	-1.3 ± 9.2	0.315
**O**_**2**_** pulse, ml·beat**^**−1**^								
T0	10.5 ± 2.8	76.5 ± 15.9		8.4 ± 2.0	80.8 ± 16.7	11.7 ± 2.5	74.1 ± 15.0	0.018
T1	11.4 ± 2.9	83.0 ± 16.6		9.1 ± 2.3	87.4 ± 19.2	12.7 ± 2.3	80.6 ± 14.6	0.028
∆	+ 0.9 ± 2.0	+ 6.5 ± 15.1	**0.001**	+ 0.7 ± 1.9	+ 6.5 ± 18.0	+ 1.0 ± 2.1	+ 6.4 ± 13.2	0.970
**VO**_**2**_**, ml·min**^**-1**^**kg**^**-1**^								
T0	12.3 ± 3.4	49.9 ± 12.3		11.6 ± 2.7	54.6 ± 12.3	12.6 ± 3.7	47.4 ± 11.5	0.001
T1	13.4 ± 3.6	54.6 ± 12.4		13.3 ± 3.3	61.6 ± 9.3	13.5 ± 3.9	50.7 ± 12.2	0.001
∆	+ 1.2 ± 3.2	+ 4.6 ± 12.2	**0.001**	+ 1.8 ± 2.7	+ 7.0 ± 11.7	+ 0.9 ± 3.4	+ 3.3 ± 12.4	0.079
**Ventilatory equivalent O**_**2**_** (VE/VO**_**2**_**)**								
T0	27.6 ± 5.3	78.9 ± 15.2		26.6 ± 4.4	75.7 ± 12.7	28.3 ± 5.7	80.7 ± 16.2	0.055
T1	26.9 ± 4.3	76.8 ± 12.3		26.8 ± 4.0	76.5 ± 11.5	26.9 ± 4.5	77.0 ± 12.9	0.827
∆	-0.7 ± 4.6	-2.1 ± 13.0	**0.050**	+ 0.3 ± 3.4	+ 0.9 ± 9.9	-1.3 ± 5.0	-3.7 ± 14.3	**0.042#**
**Ventilatory equivalent CO**_**2**_** (VE/VCO**_**2**_**)**								
T0	30.7 ± 4.7	n.a		30.2 ± 3.5	n.a	31.0 ± 5.3	n.a	0.354
T1	29.7 ± 4.1			29.4 ± 2.8		29.9 ± 4.7		0.482
∆	-1.0 ± 3.3		**0.001**	-0.8 ± 2.4		-1.1 ± 3.7		0.651
**Respiratory minute ventilation (VE), l·min**^**−1**^								
T0	33.7 ± 9.4	32.2 ± 7.6		26.9 ± 7.1	32.4 ± 7.7	37.4 ± 8.3	32.1 ± 7.6	0.783
T1	35.2 ± 8.4	34.3 ± 8.2		30.5 ± 6.7	37.0 ± 7.7	37.9 ± 8.1	32.8 ± 8.1	0.003
∆	+ 1.6 ± 9.2	+ 2.1 ± 8.8	**0.041**	+ 3.6 ± 7.0	+ 4.6 ± 8.4	+ 0.5 ± 10.1	+ 0.7 ± 8.8	**0.011#**
**Tidal volume (Vt), l**								
T0	1.7 ± 0.6	56.1 ± 20.2		1.3 ± 0.5	56.1 ± 24.6	1.9 ± 0.5	56.0 ± 17.4	0.985
T1	1.8 ± 0.6	61.8 ± 18.7		1.5 ± 0.5	65.8 ± 21.5	2.0 ± 0.5	59.5 ± 16.6	0.072
∆	+ 0.2 ± 0.4	+ 5.9 ± 21.2	**0.001**	+ 0.2 ± 0.3	+ 9.7 ± 26.8	+ 0.1 ± 0.4	+ 3.8 ± 17.1	0.154
**Breathing frequency (Bf), breaths·min**^**−1**^								
T0	21.6 ± 6.5	39.3 ± 11.8		22.4 ± 7.6	40.8 ± 14.0	21.2 ± 5.7	38.4 ± 10.3	0.293
T1	20.6 ± 6.2	37.4 ± 11.3		21.4 ± 6.7	39.2 ± 12.2	20.1 ± 5.9	36.5 ± 10.7	0.183
∆	-1.1 ± 5.2	-1.8 ± 9.5	**0.014**	-1.0 ± 5.5	-1.6 ± 10.1	-1.1 ± 5.1	-2.0 ± 9.2	0.831
**Ventilatory threshold 2 (VT2)**								
**Workload, watt**								
T0	113.7 ± 35.0	67.2 ± 19.6		98.1 ± 26.6	74.3 ± 17.6	122.6 ± 36.1	63.1 ± 19.6	0.001
T1	125.2 ± 36.9	73.8 ± 20.5		107.5 ± 29.1	81.8 ± 18.5	135.1 ± 37.3	69.2 ± 20.2	0.001
∆	+ 11.1 ± 20.6	+ 6.7 ± 12.2	**0.001**	+ 9.5 ± 16.9	+ 7.5 ± 13.1	+ 12.0 ± 22.4	+ 6.3 ± 11.7	0.573
**Heart rate, beat·min**^**−1**^								
T0	127.2 ± 20.5	76.5 ± 11.8		133.3 ± 20.1	81.6 ± 10.7	123.7 ± 20.1	73.6 ± 11.5	0.001
T1	124.9 ± 21.9	75.2 ± 12.5		132.2 ± 21.1	81.1 ± 10.7	120.8 ± 21.4	71.9 ± 12.3	0.001
∆	-2.4 ± 20.1	-1.4 ± 12.0	0.150	-1.0 ± 14.3	-0.4 ± 9.2	-3.2 ± 22.8	-1.9 ± 13.3	0.483
**O**_**2**_** pulse, ml·beat**^**−1**^								
T0	12.3 ± 4.0	89.3 ± 23.4		9.7 ± 2.3	93.9 ± 18.8	13.7 ± 4.1	86.7 ± 25.3	0.076
T1	12.6 ± 3.0	92.3 ± 17.5		10.3 ± 2.2	99.3 ± 17.1	13.9 ± 2.6	88.4 ± 16.5	0.001
∆	+ 0.3 ± 3.5	+ 3.0 ± 22.6	0.251	+ 0.6 ± 1.1	+ 5.4 ± 11.3	+ 0.2 ± 4.2	+ 1.6 ± 27.0	0.334
**VO**_**2**_**, ml·min**^**−1**^**·kg**^**−1**^								
T0	16.5 ± 4.0	67.5 ± 15.4		16.1 ± 3.4	75.9 ± 15.2	16.7 ± 4.4	62.7 ± 13.4	0.001
T1	17.4 ± 4.4	70.8 ± 16.1		17.2 ± 4.2	80.0 ± 14.3	17.5 ± 4.6	65.7 ± 14.7	0.001
∆	+ 0.9 ± 3.1	+ 3.2 ± 11.9	**0.001**	+ 1.1 ± 2.5	+ 4.1 ± 11.2	+ 0.8 ± 3.4	+ 2.7 ± 12.3	0.502
**Ventilatory equivalent O**_**2**_** (VE/VO**_**2**_**)**								
T0	32.0 ± 5.7	91.6 ± 16.3		31.7 ± 4.6	90.5 ± 13.1	32.3 ± 6.2	92.2 ± 17.8	0.556
T1	31.0 ± 4.5	76.8 ± 12.3		30.9 ± 3.8	88.1 ± 11.0	31.1 ± 4.8	88.9 ± 13.7	0.716
∆	-1.0 ± 5.0	-2.9 ± 14.2	**0.017**	-0.8 ± 3.6	-2.4 ± 10.3	-1.1 ± 5.6	-3.2 ± 16.0	0.743
**Ventilatory equivalent CO**_**2**_** (VE/VCO**_**2**_**)**								
T0	31.1 ± 5.1	n.a		30.2 ± 3.3	n.a	31.7 ± 5.9	n.a	0.107
T1	30.5 ± 4.4			29.9 ± 3.1		30.8 ± 5.0		0.204
∆	-0.6 ± 3.7		**0.038**	-0.4 ± 2.5		-0.8 ± 4.2		0.486
**Respiratory minute ventilation (VE), l·min**^**−1**^								
T0	51.3 ± 12.6	49.6 ± 11.1		43.6 ± 10.5	52.6 ± 11.5	55.6 ± 11.7	47.9 ± 10.5	0.017
T1	51.9 ± 11.6	50.5 ± 10.6		44.4 ± 9.2	54.1 ± 10.3	56.0 ± 10.7	48.5 ± 10.2	0.002
∆	+ 0.4 ± 11.8	+ 0.9 ± 11.2	0.662	+ 0.8 ± 7.6	+ 1.5 ± 9.6	+ 0.2 ± 13.6	+ 0.5 ± 12.0	0.600
**Tidal volume (Vt), l**								
T0	2.1 ± 0.6	70.7 ± 22.3		1.7 ± 0.5	74.5 ± 27.3	2.3 ± 0.5	68.6 ± 18.8	0.171
T1	2.1 ± 0.6	72.8 ± 20.8		1.8 ± 0.5	77.3 ± 23.7	2.3 ± 0.5	70.3 ± 18.6	0.071
∆	+ 0.1 ± 0.4	+ 2.3 ± 21.6	**0.028**	+ 0.1 ± 0.3	+ 2.8 ± 27.7	+ 0.1 ± 0.4	+ 2.0 ± 17.3	0.851
**Breathing frequency (Bf), breaths·min**^**−1**^								
T0	25.9 ± 6.9	47.1 ± 12.5		26.4 ± 6.9	48.0 ± 12.6	25.6 ± 6.8	46.6 ± 12.4	0.530
T1	25.1 ± 5.7	45.7 ± 10.4		25.9 ± 5.6	47.2 ± 10.1	24.7 ± 5.7	44.9 ± 10.4	0.204
∆	-0.8 ± 5.9	-1.5 ± 10.8	0.108	-0.4 ± 4.7	-0.8 ± 8.5	-1.0 ± 6.5	-1.8 ± 11.9	0.589
**Peak exercise (VO**_**2peak**_**)**								
**Respiratory exchange rate (RER)**								
T0	1.06 ± 0.1	87.7 ± 7.9		1.09 ± 0.1	89.7 ± 8.7	1.05 ± 0.1	86.6 ± 7.2	0.027
T1	1.06 ± 0.1	87.6 ± 6.7		1.08 ± 0.1	89.2 ± 8.3	1.05 ± 0.1	86.8 ± 5.4	0.062
∆	-0.0 ± 0.1	-0.1 ± 6.4	0.922	-0.0 ± 0.1	-0.5 ± 7.5	+ 0.0 ± 0.1	+ 0.2 ± 5.7	0.532
**Rating of perceived exertion (Borg Scale)**								
T0	9 (5)	n.a		9 (5)	n.a	9 (5)	n.a	0.578
T1	9 (4)			9 (4)		9 (4)		0.364
∆	0 (9)		**0.049**	0 (9)		0 (8)		0.940
**Workload, watt**								
T0	127.2 ± 36.2	74.5 ± 18.7		108.4 ± 24.9	80.1 ± 14.9	137.7 ± 37.4	71.6 ± 19.9	0.013
T1	141.4 ± 36.6	82.7 ± 19.5		121.0 ± 28.3	88.3 ± 14.2	152.9 ± 40.5	79.8 ± 21.2	0.018
∆	+ 14.2 ± 18.9	+ 8.2 ± 10.0	**0.001**	+ 12.5 ± 13.6	+ 8.2 ± 8.8	+ 15.2 ± 21.3	+ 8.3 ± 10.6	0.990
**Heart rate, beat·min**^**−1**^								
T0	133.7 ± 20.7	**80.4 ± 11.7**		140.2 ± 20.8	85.8 ± 11.1	130.1 ± 19.9	77.4 ± 10.9	0.001
T1	133.2 ± 20.8	80.2 ± 11.6		140.4 ± 20.3	86.2 ± 10.6	129.2 ± 20.0	76.8 ± 10.8	0.001
∆	-0.5 ± 14.7	-0.3 ± 8.9	0.664	+ 0.2 ± 15.1	+ 0.3 ± 9.3	-0.9 ± 14.6	-0.6 ± 8.7	0.548
**O**_**2**_** pulse, ml·beat**^**−1**^								
T0	12.6 ± 3.1	92.0 ± 16.2		10.0 ± 2.3	96.8 ± 18.4	14.1 ± 2.5	89.4 ± 14.3	0.014
T1	13.3 ± 3.3	97.2 ± 17.7		10.6 ± 2.2	102.1 ± 17.2	14.8 ± 2.7	94.4 ± 17.5	0.012
∆	+ 0.7 ± 2.1	+ 5.2 ± 14.4	**0.001**	+ 0.6 ± 1.1	+ 5.3 ± 10.8	+ 0.7 ± 2.4	+ 5.1 ± 16.0	0.917
**VO**_**2**_**, ml·min**^**−1**^**·kg**^**−1**^								
T0	18.0 ± 4.3	73.6 ± 15.0		17.4 ± 3.3	82.0 ± 14.3	18.4 ± 4.7	68.8 ± 13.3	0.001
T1	19.1 ± 4.7	77.6 ± 16.2		18.7 ± 4.0	87.1 ± 13.9	19.3 ± 5.1	72.3 ± 14.9	0.001
∆	+ 1.1 ± 2.9	+ 4.0 ± 11.0	**0.001**	+ 1.3 ± 2.4	+ 5.1 ± 10.6	+ 1.0 ± 3.1	+ 3.4 ± 11.2	0.382
**Ventilatory equivalent O**_**2**_** (VE/VO**_**2**_**)**								
T0	34.4 ± 7.0	98.1 ± 20.1		34.7 ± 6.9	99.0 ± 19.8	34.2 ± 7.1	97.6 ± 20.4	0.692
T1	33.9 ± 5.9	96.7 ± 17.0		33.7 ± 5.5	96.2 ± 15.6	34.0 ± 6.2	97.1 ± 17.7	0.761
∆	-0.5 ± 5.0	-1.4 ± 14.4	0.243	-1.0 ± 4.4	-2.9 ± 12.6	-0.2 ± 5.3	-0.6 ± 15.3	0.360
**Ventilatory equivalent CO**_**2**_** (VE/VCO**_**2**_**)**								
T0	32.4 ± 5.7	n.a		31.8 ± 4.4	n.a	32.7 ± 6.4	n.a	0.354
T1	31.9 ± 5.0			31.1 ± 3.4		32.4 ± 5.7		0.144
∆	-0.4 ± 3.7		0.165	-0.7 ± 2.8		-0.3 ± 4.2		0.602
**Respiratory minute ventilation (VE), l·min**^**−1**^								
T0	59.8 ± 14.1	57.9 ± 13.3		**0.016**	62.2 ± 14.4	64.6 ± 13.3	55.5 ± 12.0	0.005
T1	62.2 ± 15.6	60.4 ± 14.1		**0.016**	64.4 ± 13.5	64.6 ± 13.3	58.2 ± 14.1	0.010
∆	+ 2.4 ± 11.7	+ 2.5 ± 10.9	**0.016**	+ 1.6 ± 8.5	+ 2.2 ± 10.6	+ 2.8 ± 13.1	+ 2.7 ± 11.2	0.797
**Tidal volume (Vt), l**								
T0	2.1 ± 0.6	73.0 ± 22.0		1.8 ± 0.4	76.9 ± 27.8	2.4 ± 0.5	70.8 ± 17.7	0.160
T1	2.2 ± 0.6	75.4 ± 19.9		1.8 ± 0.4	79.0 ± 21.5	2.5 ± 0.5	73.4 ± 18.8	0.117
∆	+ 0.1 ± 0.3	+ 2.5 ± 20.6	**0.002**	+ 0.1 ± 0.2	+ 2.1 ± 25.7	+ 0.1 ± 0.3	+ 2.8 ± 17.2	0.860
**Breathing frequency (Bf), breaths·min**^**−1**^								
T0	28.9 ± 6.6	52.6 ± 12.0		30.0 ± 7.0	54.6 ± 12.6	28.3 ± 6.4	51.5 ± 11.6	0.147
T1	28.9 ± 6.9	52.5 ± 12.5		29.9 ± 6.2	54.5 ± 11.2	28.3 ± 7.3	51.4 ± 13.1	0.155
∆	-0.0 ± 5.2	-0.0 ± 9.3	0.936	-0.1 ± 4.4	-0.0 ± 8.0	-0.0 ± 5.6	-0.0 ± 10.0	0.986
**Breathing reserve (BR), %**								
T0	39.3 ± 17.6	n.a		31.6 ± 19.0	n.a	43.6 ± 15.1	n.a	0.001
T1	39.0 ± 16.8			35.6 ± 15.1		43.6 ± 15.1		0.066
∆	39.0 ± 16.8		0.810	+ 4.0 ± 13.9		-2.7 ± 16.4		**0.014#**

Data is presented as mean ± SD or median (range), at admission (T0) and discharge (T1) with respective changes (delta). If indicated, percent of predicted values adjusted for sex, age and body surface area are provided. Differences between groups over time were analyzed using two-way repeated measures ANOVA

^#^ Significant time × group interaction

**Table 3:** Changes in pulmonary function assessed by spirometry

	**Overall** **N=145**		p**-value time**	**Female** **N=52**		**Male** **N=93**		p**-value** **group**
	**absolut**	**% predicted**		**absolut**	**% predicted**	**absolut**	**% predicted**	
**Spirometry**								
**Vital capacity (VC), l**								
T0	4.1 ± 1.0	96.8 ± 15.6		3.3 ± 0.6	101.1 ± 17.1	4.6 ± 0.8	94.5 ± 14.3	0.019
T1	4.1 ± 0.9	97.9 ± 14.1		3.3 ± 0.6	102.7 ± 15.6	4.6 ± 0.7	95.2 ± 12.6	0.004
∆	+0.0 ± 0.4	+1.0 ± 9.7	0.244	+0.1 ± 0.4	+1.6 ± 11.4	+0.0 ± 0.4	+0.7 ± 8.6	0.640
**Forced expiratory volume in 1 second (FEV1), l**								
T0	3.0 ± 0.9	87.8 ± 20.7		2.3 ± 0.7	81.5 ± 22.2	3.4 ± 0.8	91.4 ± 19.0	0.009
T1	3.0 ± 0.8	89.7 ± 18.6		2.4 ± 0.6	87.2 ± 20.2	3.4 ± 0.7	91.1 ± 17.6	0.239
∆	+0.0 ± 0.6	+1.9 ± 17.3	0.353	+0.2 ± 0.5	+5.6 ± 15.9	+0.0 ± 0.7	-0.2 ± 17.8	0.051
**Forced expiratory volume in 1 second/vital capacity (FEV1/VC), % **								
T0	72.4 ± 12.9	92.2 ± 16.6		69.0 ± 15.1	86.3 ± 18.7	74.3 ± 11.2	95.5 ± 14.4	0.003
T1	73.3 ± 11.8	93.3 ± 15.0		72.9 ± 12.8	91.1 ± 16.0	73.5 ± 11.2	94.5 ± 14.3	0.203
∆	+0.9 ± 11.9	+1.1 ± 15.0	0.382	+3.9 ± 11.3	+4.8 ± 14.0	-0.8 ± 12.0	-1.0 ± 15.2	**0.027#**
**Peak expiratory flow (PEF), l·sec**^**-1**^								
T0	5.2 ± 2.4	63.3 ± 26.1		3.8 ± 1.8	56.3 ± 25.7	6.0 ± 2.3	67.2 ± 25.7	0.016
T1	5.6 ± 2.3	69.3 ± 26.1		4.3 ± 1.7	64.1 ± 24.9	6.4 ± 2.3	72.2 ± 26.4	0.072
∆	+0.5 ± 2.0	+6.0 ± 23.8	**0.005**	+0.5 ± 1.4	+7.8 ± 21.0	+0.5 ± 2.3	+5.1 ± 25.3	0.507
**Maximum expiratory flow at 75% of forced expiratory vital capacity (MEF75), l·sec**^**-1**^								
T0	4.7 ± 2.2	66.4 ± 27.2		3.5 ± 1.6	60.1 ± 26.9	5.5 ± 2.1	69.9 ± 26.9	0.038
T1	5.0 ± 2.2	70.5 ± 28.3		3.9 ± 1.6	67.7 ± 27.7	5.6 ± 2.2	72.0 ± 28.7	0.387
∆	+0.3 ± 1.8	+4.1 ± 25.5	0.091	+0.4 ± 1.3	+7.6 ± 23.3	+0.2 ± 2.1	+2.1 ± 26.6	0.211
**Maximum expiratory flow at 50% of forced expiratory vital capacity (MEF50), l·sec**^**-1**^								
T0	3.6 ± 1.6	78.0 ± 33.3		2.7 ± 1.3	65.3 ± 31.5	4.1 ± 1.5	85.2 ± 32.3	0.001
T1	3.6 ± 1.7	79.8 ± 34.7		2.9 ± 1.3	72.7 ± 31.3	4.0 ± 1.7	83.7 ± 36.0	0.067
∆	+0.1 ± 1.3	+1.7 ± 28.2	0.566	+0.3 ± 1.1	+7.4 ± 26.0	-0.1 ± 1.4	-1.4 ± 29.1	0.071
**Maximum expiratory flow at 25% of forced expiratory vital capacity (MEF25), l·sec**^**-1**^								
T0	1.7 ± 0.7	89.3 ± 34.0		1.3 ± 0.6	76.8 ± 32.9	1.9 ± 0.7	96.3 ± 32.8	0.001
T1	1.6 ± 0.7	87.6 ± 34.9		1.3 ± 0.6	80.3 ± 34.2	1.8 ± 0.7	91.6 ± 34.8	0.060
∆	+0.0 ± 0.6	-1.7 ± 29.9	0.358	+0.1 ± 0.5	+3.4 ± 26.9	-0.1 ± 0.6	-4.6 ± 31.2	0.118

Data is presented as mean ± SD, at admission (T0) and discharge (T1) with respective changes (Delta). If indicated, percent of predicted values adjusted for sex, age and body surface area are provided. Differences between groups over time were performed using two-way repeated measures ANOVA

^#^ Significant time × group interaction

#### Perceived disease burden and workability

**Fig. 3:**
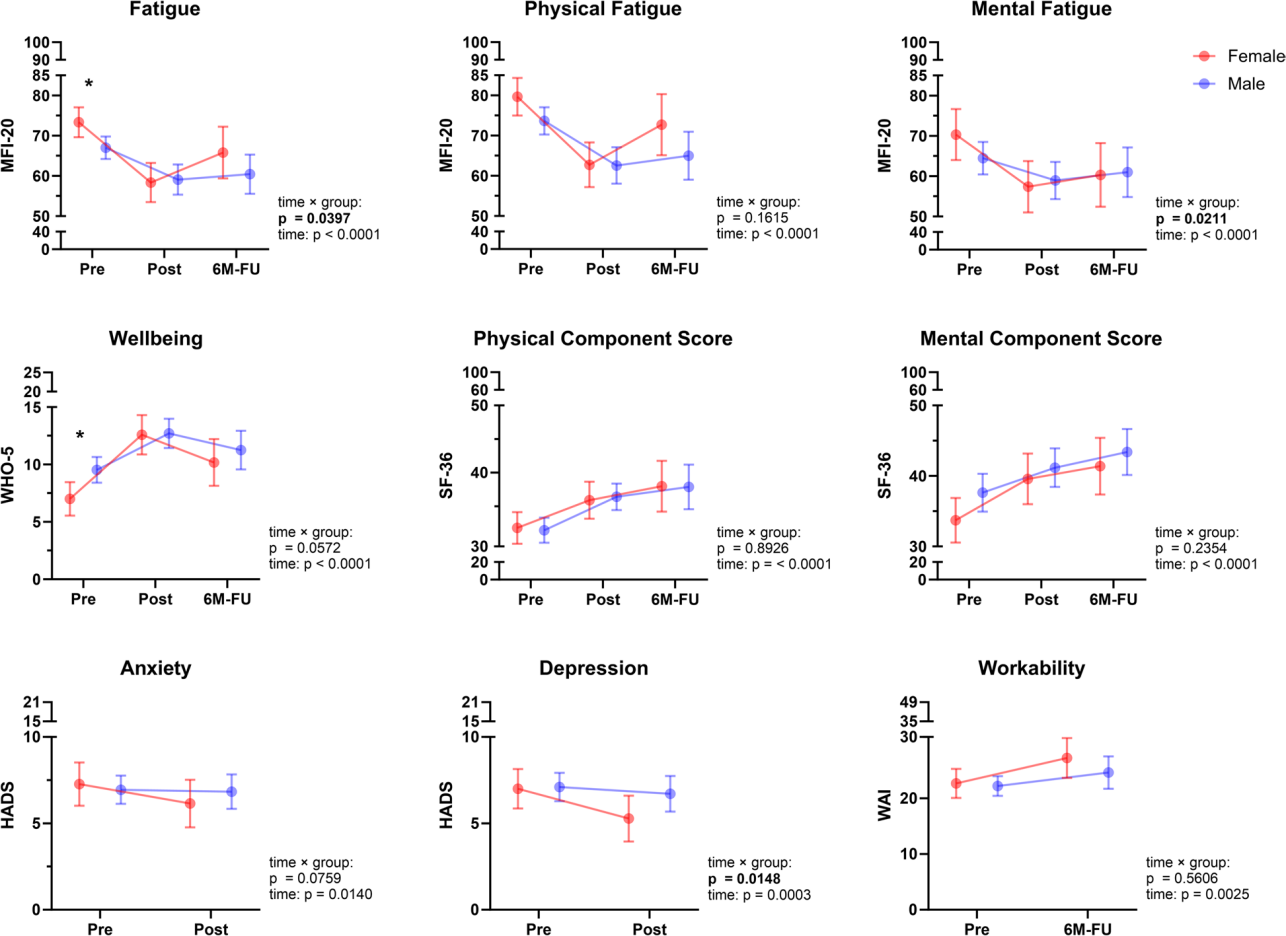
Female and male patients with Post-COVID-19 syndrome (PCS) differ in disease perception. Women presented with significantly higher levels of fatigue and lower wellbeing compared to men. While subjective impact of disease was significantly improved in all domains over time (time effect), female PCS patients showed a significantly different time course for development of fatigue and depression (significant time × group interaction). Disease perception was assessed by validated questionnaires at admission (Pre), discharge (Post) and after 6-months (6 M-FU). Anxiety, depression (pre, post) and workability (pre, 6 M-FU) were only assessed twice. Questionnaires were completed at admission (*N* = 132), discharge (*N* = 128) and 6-month follow-up (*N* = 89). Data is presented as mean and 95% CI. Differences between groups over time were performed using mixed-effects model. MFI-20, Multidimensional Fatigue Inventory; WHO-5, Well-being; SF-36, Health-related quality of life; HADS, Hospital Anxiety Depression Scale; WAI, Workability Index. * *p* ≤ 0.05

PCS patients showed overall high levels of fatigue (MFI-20 score, 69.2 ± 13.2) and low health-related quality of life for both, the physical (31.5 ± 8.3) and mental (36.2 ± 11.8) SF-36 components at admission (Fig. 3). Fatigue showed considerable negative correlations with exercise parameters peak workload (*p* = 0.040, *r* = -0.179), VE (*p* = 0.027, *r* = -0.192) and tidal volume (*p* = 0.041, r = -0.178), as well as pulmonary function (FEV1/VC, *p* = 0.007; *r* = -0.235; PEF, *p* = 0.032, r = -0.186; MEF50, *p* = 0.013; *r* = -0.215) (Additional file 1: Figure S1). Overall workability was low (22.3 ± 7.8) with a median maximum incapacity for work of 99 days during the last 12 months, while wellbeing was significantly reduced. Notably, female patients demonstrated significantly higher levels of fatigue and lower ratings of wellbeing at baseline (MFI-20 score, 73.4 ± 12.5; WHO-5 score, 7.0 ± 5.0) compared to males (MFI-20 score, 67.0 ± 13.1; WHO-5 score, 9.5 ± 5.2, both *p* ≤ 0.027). Of note, ~ 60% of female patients had an MFI-20 score above 70 (*n* = 29).

### Rehabilitation and rehabilitation outcomes

The overall mean length of inpatient rehabilitation was ~ 4 weeks (28.8 ± 6.1 days) with no significant difference between female and male patients (*p* = 0.401). During rehabilitation, patients performed a mean of 12 exercise-based (active) therapy sessions per week with a participation rate of 91.4 ± 13.6% (female 90.2 ± 12.1%; male, 92.0 ± 14.5%; *p* = 0.437). Of these, 3–4 sessions were ergometer training, in which female and male patients were able to increase their workload equally by ~ 10%. Occurrence of post exercise malaise (PEM) was very low with only 3 reported cases. Overall, patients performed 1586.8 ± 479.4 min of exercise (cardio, 1248.3 ± 398.4 min; strength, 338.5 ± 145.3 min) equal to 107.0 ± 37.0 METs (female, 100.2 ± 36.2 METs; male, 110.7 ± 37.2 METs; *p* = 0.103), which were comparable between female and male patients in terms of cardio METs (female, 81.6 ± 31.5 METs; male, 90.4 ± 34.1 METs; *p* = 0.131) and strength METs (female, 18.6 ± 9.5 METs; male, 20.4 ± 7.9 METs; *p* = 0.262). In addition, patients performed ~ 8 educational, counselling, and occupational therapies and ~ 3 relaxation therapies per week.

**Fig. 4:**
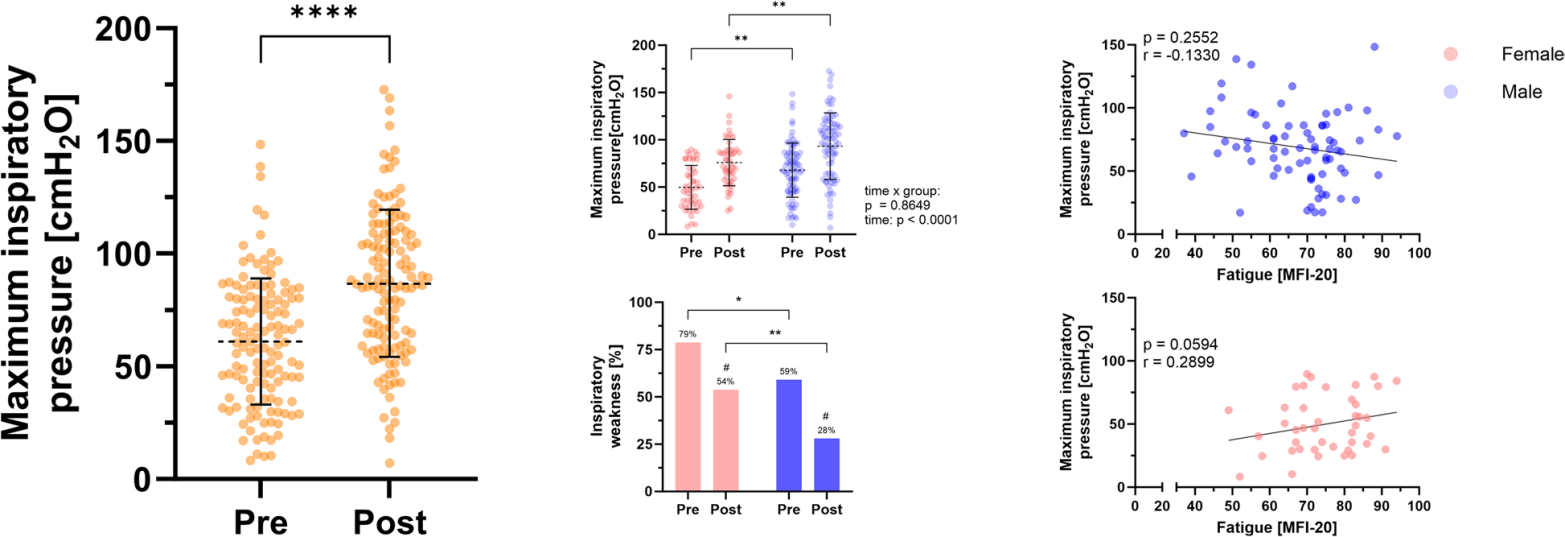
Patients with Post-COVID-19 syndrome (PCS) showed improved maximum inspiratory pressure (MIP) in response to rehabilitation. MIP was assessed at admission (Pre) and discharge (Post). Inspiratory weakness (MIP ≤ 80 cmH2O) was detected in 66.2% of PCS patients at admission and 37.2% of PCS patients at discharge, differing significantly between female and male patients. In female patients, higher MIP was associated with higher fatigue, while male patients showed lower MIP with decreasing fatigue. Data is presented as mean ± SD, each data point represents an individual measurement. Differences between groups over time were performed using two-way repeated measures ANOVA. Between-group comparison was performed using Chi-square test. Within-group comparison was performed using Mc-Nemar-Test. Correlations were performed using Spearman rank correlation. * *p* ≤ 0.05, ** *p* ≤ 0.01, **** p ≤ 0.0001, # Significantly different from Pre to Post (*p* ≤ 0.001)

During rehabilitation, an overall improvement of physical exercise capacity was detected at submaximal and peak load (all *p* ≤ 0.001; Fig. 2) in that peak oxygen uptake increased by 1.1 ± 2.9 ml/min/kg (4.6 ± 12.2% reference) while oxygen uptake at VT1 and VT2 increased by 1.2 ± 3.2 ml/min/kg (4.0 ± 11.0%) and 0.9 ± 3.1 ml/min/kg (+ 3.2 ± 11.9%), respectively. In addition, pre-exercise resting heart rate was significantly lower, while O_2_-pulse, respiratory minute volume and tidal volume at both submaximal and peak exercise increased significantly (all *p* ≤ 0.041; Table 2). Notably, and despite male patients starting at a lower level of cardiopulmonary exercise capacity, female patients showed greater improvements for workload at VT1 (time x group interaction, *p* = 0.0262) and a tendency to greater improvements in oxygen uptake (not significant, *p* = 0.079). Since men and women were not matched for cardiopulmonary exercise capacity, ANCOVA with baseline values for oxygen uptake as covariates was performed. This analysis suggested that female and male patients differed significantly in improvement of oxygen uptake at VT1 and peak load (both *p* ≤ 0.013). In addition, female patients showed higher improvements in respiratory minute ventilation at submaximal exercise (female, + 4.6 ± 8.4%; male, + 0.7 ± 8.8%; time x group interaction, *p* = 0.011). Application of more stringent criteria for maximal exercise testing (RER ≥ 0.95 for both CPETs, *N* = 128) revealed similar results. Responder analysis suggested that female and male patients responded equally to exercise-based rehabilitation at submaximal load (female, 67%; male 59%, *p* = 0.331) and at peak exercise (female, 60%; male, 58%; *p* = 0.856). Analyses of pulmonary function revealed an overall improvement for peak expiratory flow of 0.5 ± 2.0 l/s equal to 6.0 ± 23.8% (*p* = 0.005). Notably, female patients showed greater improvements for FEV1/VC compared to men (female, 4.8 ± 14.0; male, -1.0 ± 15.2; time x group interaction, *p* = 0.027), however, adjustment for baseline values did not confirm this difference (*p* = 0.590). Overall, MIP was significantly increased from 61.1 ± 28.1 cmH_2_O to 86.8 ± 32.6 cmH_2_O (*p* < 0.001; Fig. 4) with no significant difference between female and male patients (*p* = 0.865). However, 53.8% of female patients and only 28.0% of male patients were discharged with inspiratory weakness (≥ 80 cmH_2_O) (*p* = 0.004). Of note, patients were able to reduce their body weight by ~ 1.5 kg during rehabilitation (*p* < 0.001).

In terms of disease perception, patients demonstrated improvements in fatigue, health-related quality of life, including the subcategories physical and mental component as well as wellbeing, depression and anxiety (all *p* ≤ 0.014; Fig. 3). Notably, female patients showed greater improvements in overall fatigue (female, -16.0 ± 15.8; male -7.5 ± 14.1), mental fatigue (female, -14.8 ± 17.6; male, -5.4 ± 13.2) and depression scores (female, -1.7 ± 3.8; male, -0.3 ± 2.5) compared to males (all time x group interaction: *p* ≤ 0.040). Of note, differences between female and male patients in improvement of disease perception, as well as exercise capacity and pulmonary function (except FEV1/VC) were independent of age, baseline body mass index, comorbidities and baseline values.

### Maintenance effects after 6 months

To record the medium-term effects of rehabilitation on disease perception and workability, patients were asked to fill out questionnaires after 6 months. Health-related quality of life as well as overall and mental fatigue remained unchanged compared to discharge, while patients showed a significant re-increase of physical fatigue (+ 4.6 ± 20.9; *p* = 0.032, 6 M-FU vs. post), with greater worsening in women (female, + 9.4 ± 23.7; male, + 1.7 ± 18.6; not significant, *p* = 0.129). In addition, wellbeing significantly decreased after discharge (-1.9 ± 6.0; *p* = 0.007) while workability showed an increase from admission to 6-months follow-up (*p* = 0.003) with no significant differences between men and women.

## Discussion

This study evaluated the efficacy of inpatient exercise-based medical rehabilitation in female and male patients with long-term Post-COVID-19 Syndrome (PCS) in terms of pulmonary function, physical exercise capacity and perceived disease burden. In brief, the key findings of this study are (1) female patients presented to rehabilitation with higher exercise performance capacity, lower pulmonary function and elevated perceived disease burden compared to male patients, (2) while overall exercise capacity was significantly enhanced in women and men, female PCS patients showed larger improvements in absolute and relative submaximal performance, (3) expiratory flow limitation in terms of forced expiratory volume (FEV) showed greater improvements in women compared to men, while (4) inspiratory weakness was still present in a greater proportion of women (50%) compared to men (30%) at discharge.

PCS patients in this study were referred to medical rehabilitation with a clinically relevant reduction in submaximal and peak oxygen uptake, a finding that has already been observed in comparable cohorts [30, 31]. Of note, this overall limitation in physical capacity at ~ 75% reference is comparable to cardiologic patients referred to medical rehabilitation after myocardial infarction and subsequent interventions, despite the fact that PCS patients are significantly younger [32]. Recent studies investigated the efficiency of exercise-based rehabilitation programs (8-weeks, 3 supervised multicomponent exercise sessions per week, outpatient program) in PCS patients suggesting improvements on physical performance ranging from 2.1 ml/min/kg to 2.5 ml/min/kg in maximum oxygen uptake as well as improved quality of life and reduced fatigue and depression in non-hospitalized PCS patients [13, 14]. While these studies provide evidence that physical exercise training as part of medical rehabilitation can improve exercise capacity in PCS patients with mild to moderate symptom severity, none of the existing studies reported on analyses of potential differences between female and male PCS patients, even though the proportion of women was considerably high (~ 70%) [13, 14]. Data from our medical rehabilitation center suggests that female PCS patients are 5 years younger on admission, had been hospitalized less frequently, and need for ventilation during acute care was lower compared to male patients. At the start of rehabilitation, restrictions in physical exercise capacity were milder in women, while limitations in pulmonary function including FEV, PEF, MEF and MIP were greater. It is one major finding of the present study that male PCS patients, despite presenting with significantly lower cardiopulmonary exercise capacity at rehabilitation start, do not show greater improvement in physical fitness than female PCS patients and still differ from their female counterparts upon discharge.

Recent studies showed that patients recovered from a COVID-19 infection suffer from persistently impaired pulmonary and respiratory muscle function [33, 34]. While most studies do not report findings by sex, a study on respiratory muscle dysfunction approximately 5 months after acute infection reported that inspiratory muscle weakness was more frequent in women (up to ~ 96%) compared to men suffering from long-COVID [35], in line with our observations. We also provide evidence that exercise-based rehabilitation including inspiratory muscle training may improve inspiratory muscle weakness, a finding previously reported from patients after a COVID-19 infection with the lead symptom of breathlessness [36]. Notably, this study [36] enrolled predominantly women (88%) reporting a mean MIP of ~ 80 cmH_2_O, which is somewhat higher compared to ~ 50 cmH_2_O detected in our female participants.

We also detected significant differences in disease burden as fatigue was significantly more pronounced in women while wellbeing was lower compared to men at rehabilitation start. Of note, this observation can only partially be explained by reduced relative submaximal and maximal oxygen uptake capacity, which was considerably more restricted in men. By contrast, studies in individuals with mild-to-moderate disability multiple sclerosis suggested, that significant correlations between expiratory muscle strength, expiratory pressure and fatigue exist [37]. Thus, greater limitations in pulmonary and respiratory muscle function in female long-term PCS patients may be related to the observed high levels of fatigue. In this regard, it is of interest that women reported a greater improvement in fatigue during rehabilitation compared to men. Notably, this was accompanied by a larger improvement of FEV over time. In terms of maintenance effects, fatigue, wellbeing, health-related quality of life and depression appeared stable at the ameliorated post-rehabilitation level. However, fatigue in female PCS patients showed a trend towards re-elevation. This observation indicates that even longer post-rehabilitation screenings of PCS relapse may be indicated. Since data on the long-term effects of rehabilitation over the course of 6 months is scarce, it is unclear if the observed improvements in health-related quality of life including physical and mental components as well as improvements of fatigue and workability can be achieved by rehabilitation programs focusing less on physical exercise training and more on pulmonary rehabilitation. Future studies should also consider that the widely-used SF-36 questionnaire indicated a trend to further improvements over the 6 months post-rehabilitation period while the results of the MFI-20 screener indicated deviations towards re-elevated fatigue levels.

Since the PCS complex is still not well-understood, underlying causes and mechanisms for the observed differences between female and male PCS patients are hypothetical. One possible explanation may lie in sex-dependent physiological differences of serotonin levels, which are higher in men than in women and may contribute to fatigue and disease perception. Recently, PCS was associated with a reduction of serotonin levels, potentially driven by viral RNA-induced type I interferons [38]. The serotonin approach combines several hypotheses to explain the etiology of PCS, including viral persistence, chronic inflammation, hypercoagulability and autonomic dysfunction [38], the latter also recently described in our patients [39].

Some limitations to the present study may exist. Women were significantly younger at onset of PCS and admission to rehabilitation. While we accounted for this difference by using reference values corrected for sex, age and body surface area, other differences such as prevalence of comorbidities should be interpreted with care. Even if ANCOVA revealed no effect of comorbidities on the observed differences, men were more frequently affected by predominantly cardiovascular conditions, which may have affected the effectiveness of rehabilitation in male PCS patients. It needs to be considered that the application of stringent objective criteria for maximal exercise testing is difficult in PCS patients. The reported findings for peak cardiopulmonary fitness thus need to be interpreted with care. It might be preferrable to apply submaximal exercise tests in PCS populations, also with respect to the here reported findings in sex-specific differences in submaximal performance changes. PCS patients enrolled in this study suffered from long-term symptom persistence, patients were capable of participating in a medical rehabilitation program as described. Our findings may not be transferred to PCS patients with greater symptom severity or different organ manifestations.

## Conclusions

We conclude that restrictions in cardiopulmonary fitness and pulmonary function can be ameliorated by exercise-based medical rehabilitation in female and male PCS patients. Improvements are associated with increased health-related quality of life and well-being, reduction in fatigue and depression and higher workability also over a time-span of 6 months post-rehabilitation. Of note, female and male PCS patients referred to rehabilitation differ in terms of fatigue, physical fitness and pulmonary function, indicating that sex-specific examinations and therapies are needed for a tailored rehabilitation program and individual optimization of outcome. Initial clinical assessment should include CPET and pulmonary function tests as well as assessments of fatigue for all PCS patients. Women with PCS might benefit from intensified respiratory (muscle) training, while adjusted aerobic exercise training may lead to greater improvements of cardiopulmonary fitness in men. Further studies are required to investigate the mechanisms leading to the observed differences in pulmonary limitations and physical performance deficits in women and men with PCS.

## Supplementary information

Additional file 1. Figure S1 – Correlations between exercise capacity, pulmonary function and disease perception.

## Data Availability

Datasets used in this study are available from the corresponding author upon reasonable request.

## Abbreviations

6 M-FU: 6 Month follow-up
BMI: Body mass index
CI: Confidence interval
CPET: Cardiopulmonary exercise testing
FEV1: Forced expiratory volume in 1 s
HADS: Hospital Anxiety Depressions Scale
HR: Heart rate
MEF: Maximum expiratory flow of forced expiratory vital capacity
MET: Metabolic equivalent
MFI-20: Multidimensional Fatigue Inventory
MIP: Maximum inspiratory pressure
PCS: Post-COVID-19 Syndrome
PEF: Peak expiratory flow
PEM: Post exercise malaise
RER: Respiratory exchange rate
RPE: Rating of perceived exertion
SD: Standard deviation
SF-36: 36-Item Short Form Survey Health-related Quality of Life
TE: Typical error
VC: Vital capacity
VCO_2_: Carbon dioxide production
VE: Minute ventilation
VO_2_: Oxygen consumption
VT1: First ventilatory threshold
VT2: Second ventilatory threshold
W: Workload
WAI: Work Ability Index
WHO-5: WHO-5 wellbeing index
