# Supplementary material for: Sex-specific differences of cardiopulmonary fitness and pulmonary function in exercise-based rehabilitation of patients with long-term post-COVID-19 syndrome
Source: BMC Med. 2024 Oct 8;22:446. doi: 10.1186/s12916-024-03658-8 (PMC11463035; doi:10.1186/s12916-024-03658-8)
Supplement: Supplementary file 1 — Additional file 1. Figure S1 – Correlations between exercise capacity, pulmonary function and disease perception. [file 12916_2024_3658_MOESM1_ESM.pdf]

Additional File: Supplemental Figure 1

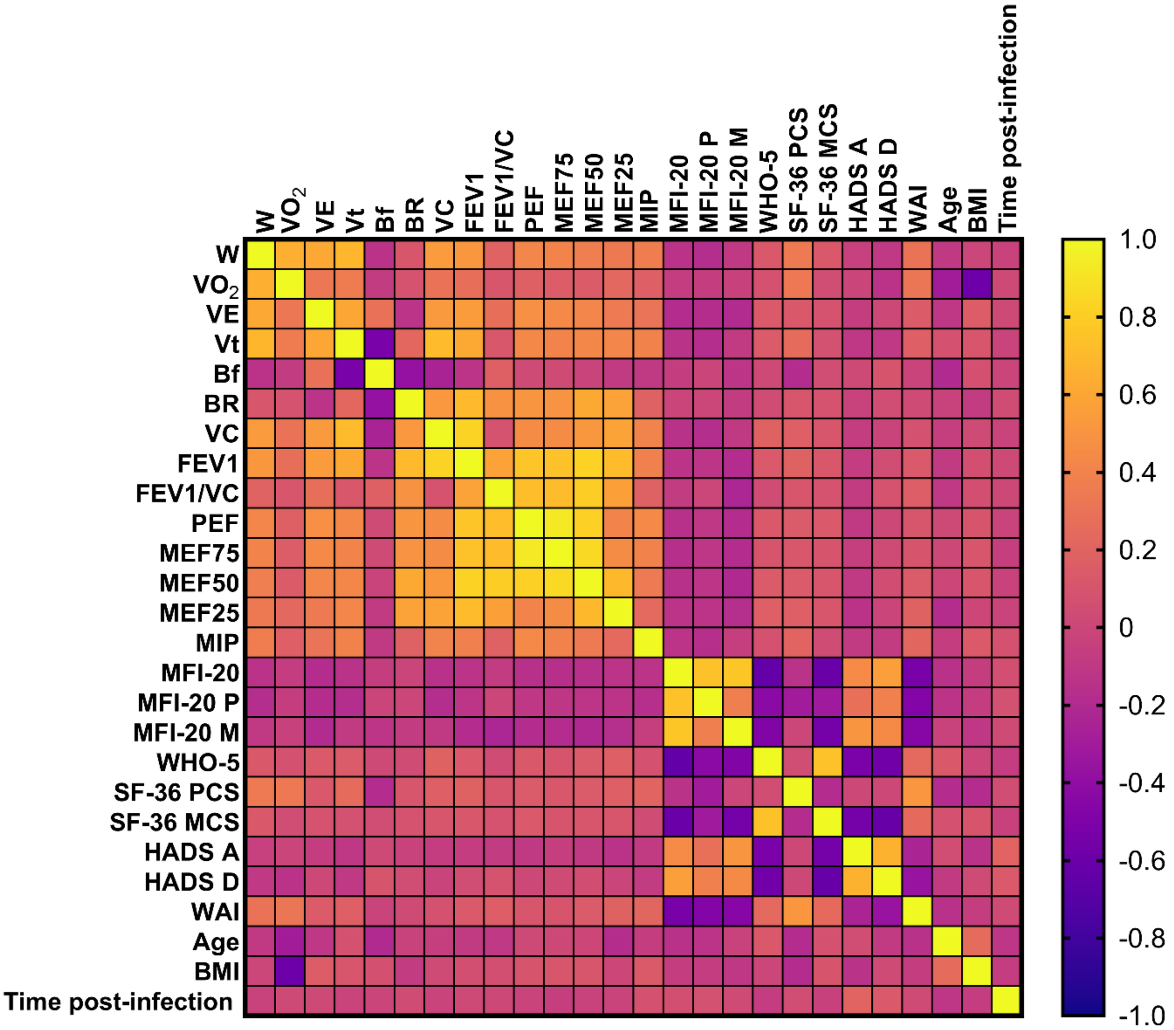

Figure S1: Patients with Post-COVID-19 syndrome (PCS) showed reduced exercise capacity and pulmonary function as well as increased perceived disease burden on admission, with variations in severity depending on sex. Correlations were predominantly detected between fatigue (especially the mental component) and parameters of CPET (W, VE, Vt; all $p \leq 0.041$) and pulmonary function (VC, FEV1, FEV1/VC, PEF, MEF; all $p \leq 0.043$). No correlations were found with time after infection. Multivariate matrix was created using Spearman rank correlation.
